# In silico and *in vitro* evaluation of antiviral activity of wogonin against main protease of porcine epidemic diarrhea virus

**DOI:** 10.3389/fcimb.2023.1123650

**Published:** 2023-03-15

**Authors:** Jieru Wang, Xiaoyu Zeng, Dongdong Yin, Lei Yin, Xuehuai Shen, Fazhi Xu, Yin Dai, Xiaocheng Pan

**Affiliations:** ^1^ Anhui Province Key Laboratory of Livestock and Poultry Product Safety Engineering, Livestock and Poultry Epidemic Diseases Research Center of Anhui Province, Key Laboratory of Pig Molecular Quantitative Genetics of Anhui Academy of Agricultural Sciences, Institute of Animal Husbandry and Veterinary Sciences, Anhui Academy of Agricultural Sciences, Hefei, Anhui, China; ^2^ College of Animal Science and Technology, Anhui Agricultural University, Hefei, China

**Keywords:** porcine epidemic diarrhea virus (PEDV), wogonin, main protease, antiviral activity, binding affinity

## Abstract

The high mortality rate of weaned piglets infected with porcine epidemic diarrhea virus (PEDV) poses a serious threat to the pig industry worldwide, demanding urgent research efforts related to developing effective antiviral drugs to prevent and treat PEDV infection. Small molecules can possibly prevent the spread of infection by targeting specific vital components of the pathogen’s genome. Main protease (Mpro, also named 3CL protease) plays essential roles in PEDV replication and has emerged as a promising target for the inhibition of PEDV. In this study, wogonin exhibited antiviral activity against a PEDV variant isolate, interacting with the PEDV particles and inhibiting the internalization, replication and release of PEDV. The molecular docking model indicated that wogonin was firmly embedded in the groove of the active pocket of Mpro. Furthermore, the interaction between wogonin and Mpro was validated in silico *via* microscale thermophoresis and surface plasmon resonance analyses. In addition, the results of a fluorescence resonance energy transfer (FRET) assay indicated that wogonin exerted an inhibitory effect on Mpro. These findings provide useful insights into the antiviral activities of wogonin, which could support future research into anti-PEDV drugs.`

## 1 Introduction

Porcine epidemic diarrhea (PED), an acute intestinal infectious disease caused by the porcine epidemic diarrhea virus (PEDV), results in huge economic losses for the swine industry worldwide (Zhang et al., 2020). The efficiencies of vaccines based on classic strains are significantly challenged owing to the hypervariability of PEDV, complicating field pandemics (Zhou et al., 2021). Currently, several advances have been accomplished in developing anti-PEDV drugs, and there has been a growing interest in the use of the main components of herbal extracts as suitable alternative sources for the development of antiviral agents (Gao et al., 2020). For instance, puerarin (Zhang et al., 2021; Ren et al., 2022) has been reported to inhibit PEDV replication and pro-inflammatory response, alleviating intestinal injury and modulating intestinal microorganisms in PEDV-challenged piglets (Wu et al., 2020; Wu et al., 2021), while glycyrrhizin has been noted to inhibit PEDV infection and decrease pro-inflammatory cytokine secretion *via the* HMGB1/TLR4-mitogen-activated protein kinase (MAPK) p38 pathway (Gao et al., 2020). Furthermore, monolaurin has been observed to exert a protective effect against PEDV infection in piglets by regulating the interferon pathway (Zhang et al., 2021); cinchonine has been found to suppress PEDV infection by inducing autophagy in the early stage of PEDV replication (Ren et al., 2022); and ergosterol peroxide has been reported to restrain multiple stages of the PEDV life cycle by suppressing ROS generation and the p53 signaling pathway (Liu et al., 2022). Although quercetin 7-rhamnoside did not directly interact with or inactivate PEDV particles, it affected the initial stage of PEDV infection by disturbing PEDV replication (Choi et al., 2009). Levistolide A has been demonstrated to inhibit PEDV from attaching to the cellular membrane or penetrating the cells *via* inducing ROS generation (Zeng et al., 2022). In addition, many small molecules have been noted to prevent the spread of PEDV infection by targeting specific important components of the viral genome. PEDV 3C-like protease (Mpro) plays essential roles in cleaving the majority of synthesized PEDV polyproteins, and Mpro remains the target of choice for the prophylactic or curative treatment of coronavirus (CoV) diseases owing to its high sequence and structural conservation (Ye et al., 2016; Li et al., 2020; Dampalla et al., 2022). Several flavonoids and small molecule inhibitors have been reported to inhibit the Mpro of SARS-CoV (Khan et al., 2021; Rizzuti et al., 2021), and quercetin (Li et al., 2020), barrigenol, kaempferol, myricetin (Sharma et al., 2021), a broad-spectrum inhibitor GC376 (Ye et al., 2020), and two compounds (Zhou et al., 2021) have been noted to exert strong inhibitory activity against Mpro. However, there are only a few effective commercial veterinary drugs available for the inhibition of PEDV. Consequently, there is an ever-increasing need for the development of novel drugs and therapeutic strategies for targeting Mpro.

Among the complex components of traditional Chinese medicine, “flavonoids” are a class of active components with anti-tumor, antioxidant, anti-inflammatory, antibacterial, and extensive antiviral effects, which have been widely studied (Guo et al., 2007). Wogonin, a major flavonoid, has been reported to exhibit effective anticancer and anti-inflammatory properties (Kim et al., 2013; Zhao et al., 2014) and has also been shown to exhibit direct inhibitory activity against SARS-CoV, herpes virus (HSV), respiratory syncytial virus (RSV), dengue virus (DV), H1N1 influenza virus (IV), and human immunodeficiency virus (HIV), without showing cytotoxicity (Zhi et al., 2019; Chu et al., 2020; Zhi et al., 2020). However, the effect of wogonin on PEDV infection has not yet been reported. In this study, we investigated the effects of wogonin on the PEDV infection cycle and its docking with Mpro against PEDV infection. The results provide novel insights into the potential use of wogonin as an anti-PEDV drug, which can be beneficial for the swine industry.

## 2 Materials and methods

### 2.1 Cell culture and virus

Vero and porcine small intestinal epithelial cell line (IPEC-J2) cells were maintained in Dulbecco’s modified eagle medium (DMEM; Gibco, Grand Island, NE, USA) containing 10% (v/v) fetal cattle serum (FBS), 100 U/mL penicillin, and 100 μg/mL streptomycin mixtures at 37°C with 5% CO_2_. PEDV isolate AH2012/12 (GenBank accession KU646831) was donated by researcher Li Bin of the Jiangsu Academy of Agricultural Sciences.

### 2.2 Cytotoxicity assay

Cell viability was measured using the cell counting kit-8 (CCK8) (Meibio, Beijing, China) assay, following the manufacturer’s instructions. The Vero and IPEC-J2 cells were seeded into 96-well plates containing wogonin (6.25–400 μM). Subsequently, the cells were incubated with wogonin for 48 h and then washed thrice with PBS. The culture supernatant in each well was replaced with 110 µL of DMEM containing 9.09% CCK-8 reagent and incubated in the dark for 2 h. After incubation, the cells were gently shaken, and the optical density (OD450) of the culture was measured using a microplate reader. Cell viability (CC50) was calculated as follows: cell viability = (As − Ab) ÷ (Ac − Ab) × 100%, As indicates the OD450 value of the cells incubated with wogonin and CCK-8 solution, Ab denotes the OD450 values of the wells without Vero cells, incubated with wogonin and CCK-8 solution, and Ac represents the OD450 values of the cells incubated without wogonin but with CCK-8 solution.

### 2.3 Viral infection

The Vero and IPEC-J2 cells were grown to approximately 80%–90% confluence in 6-well culture plates in the absence or presence of wogonin (12.5–100 μM) and infected with PEDV at 0.01 MOI (Vero) and 0.1MOI (IPEC-J2) with 5 μg/mL trypsin (Invitrogen) for 2 h. After washing the unbound virus with PBS, the cells were cultured in fresh medium in the absence or presence of wogonin (12.5–100 μM) with 5 μg/mL of trypsin at 37°C for 48 h until the cytopathic effect (CPE) became visible. Then, the cells were collected for viral RNA detection by real-time reverse transcription PCR (RT-qPCR), immunofluorescence assay (IFA), and a median tissue culture infectious dose (TCID50) assay. The wogonin concentration that achieved 50% protection was defined as the 50% inhibitory concentration (IC50). To calculate the IC50 values, the results were transformed to a percentage of the controls, the IC50 values were obtained from the RT-qPCR results, and the percent protection achieved by wogonin in the PEDV-infected cells was calculated. The therapeutic index was defined as CC50/IC50.

### 2.4 Effect of wogonin on PEDV life cycle

To determine the effect of wogonin on viral attachment, the Vero cells were infected with PEDV in the absence or presence of wogonin (50 μM) at 0.05 MOI at 4°C for 1 h. After washing 3 times with PBS, the cell lysates were harvested for RT-qPCR assay. For the viral internalization assay, the Vero cells were incubated with PEDV (MOI = 0.05) for 1 h at 4°C. After the unbound viruses were removed by washing thrice in PBS, the cells were treated with wogonin (50 μM) at 37°C for 1 h. The cell lysates were harvested for RT-qPCR assay. To investigate the effect of wogonin on PEDV replication, the Vero cells were infected with PEDV (MOI = 0.05) for 1 h at 37°C. Then, the cells were washed with PBS to remove non-internalized viruses and incubated with wogonin (50 μM) for 2 h at 37°C. The cell lysates were harvested for RT-qPCR. To determine the effect of wogonin on PEDV release, the Vero cells were infected with PEDV (MOI = 0.05) for 10 h at 37°C and then washed thrice with PBS and incubated with wogonin (50 μM) at 37°C for 12 h. The supernatants were harvested for RT-qPCR. To examine the effect of wogonin on PEDV inactivation, PEDV (MOI = 0.05) and wogonin (50 μM) were incubated together at 37°C for 2 h. Then, the mixture was washed with PBS and ultracentrifuged at 90,000 × g for 1.5 h at 4°C in 20% sucrose buffer (w/w) to purify the virions. Subsequently, the virions were resuspended in the culture medium, and the cell lysates were harvested for RT-qPCR (Liu et al., 2022).

### 2.5 Absolute RT-qPCR

The viral RNA from the cell suspensions was extracted using the Viral RNA Extraction Kit (#9766, TaKaRa, Shanghai, China), following the manufacturer’s instructions. The cDNA was obtained by RT-PCR with a PrimeScript™ RT Reagent Kit with a gDNA Eraser (TaKaRa, Shanghai, China). The absolute RT-qPCR assay used to quantify the PEDV genome was performed with RealUniversal SYBR Green Premix (Tiangen, Beijing, China). As an internal reference for the quantification of the PEDV copy numbers, the PEDV 186-fragment gene was cloned into the pMD19-T vector, and primers PEDV-186-F (5′-TACTAAGCGTAACATCCTGCC-3′) and PEDV-186-R (5′-GTAGTACCAATAACAACCGAAGC-3′) were employed.

### 2.6 TCID50 assay

To determine the TCID50 values of the PEDV, the viral stock solutions were serially diluted before inoculation with the confluent Vero cell monolayers grown in 96-well plates. The cells were washed thrice with PBS, and eight wells were inoculated with 100 µL of each dilution of wogonin. The plates were incubated at 37°C in 5% CO_2_ for 2 days. Wells with syncytium formation, the specific CPE caused by AH2012/12, were classified as PEDV-positive. The TCID50 of the PEDV was calculated using the Reed–Muench method.

### 2.7 Indirect immunofluorescence assay

The Vero cells infected with PEDV at different MOI were seeded onto a 6-well plate, fixed with 4% paraformaldehyde for 20 min at room temperature (RT), and permeabilized with 0.3% ice-cold TritonX-100 in PBS for 10 min at RT. After washing thrice with PBS, the cells were blocked with PBS containing 1% bovine serum albumin for 30 min at RT, incubated with anti-PEDV N protein antibodies (developed by our laboratory)(Shen et al., 2020) in PBS for 1 h at 37°C, and again washed thrice with PBS. Subsequently, the cells were incubated with Alexa 488-labeled anti-mouse antibody (Antgene, Wuhan, China) for 45 min at 37°C, washed thrice, treated with DAPI dihydrochloride at RT for 10 min in the dark (Beyotime Biotechnol, Shanghai, China) to stain the nuclei, and analyzed using an indirect immunofluorescence assay (IFA).

### 2.8 Construction of Mpro and wogonin

The RCSB protein data bank (www.rcsb.org,PDBcode:4XFQ) was employed to obtain the X-ray crystallographic structure of the pure protein, Mpro, by removing the water, ions, and ligand. The 3D structure of the ligand, wogonin, was first constructed using Gaussview 5.0, and the geometry optimization of wogonin was performed using Gaussian 09 with the ab initio calculation method at the HF/6-31G (d) level.

### 2.9 Molecular docking calculation

AutoDock Tools was used to establish the docking, and molecular docking was simulated by AutoDock software. The binding site was defined by a rectangular box with explicit coordinates, and the box was adjusted to the center of the primary binding site. Three-dimensional grids were set as 90×90×90 points with a grid spacing of 1 Å for the protein, Mpro. The center of the grid was regarded as the mass center, and the exhaustiveness was 20. The ligand, wogonin, was docked to the protein, Mpro, by turn, and the corresponding energy evaluation was also generated. The most common and stable docking conformation with the lowest docking energy was selected as the initial binding mode for the binding mode analysis.

### 2.10 Differential scanning fluorimetry

N terminal GST-tagged Mpro expression and purification were performed as described previously (Li et al., 2020) and then digested with PreScission Protease (Beyotime, Shanghai, China) and loaded onto a Glutathione Sepharose 4B column to remove the GST tag and GST-tagged PreScission Protease. A total of 1 μg/μL purified Mpro with 40 μM wogonin was subjected to nano differential scanning fluorimetry (DSF) (Prometheus NT.48) to measure the stability of Mpro, and the results were analyzed by PR. ThermControl software. Mpro and 1% DMSO PBS were used as the control, and the temperature was increased from 25°C to 95°C.

### 2.11 Microscale thermophoresis assay

The binding of Mpro to wogonin was examined using a microscale thermophoresis (MST) assay. The concentration of red-labeled Mpro was maintained at 200 nM, while that of the unlabeled binding partner (wogonin) varied in the range of 40 μM-1.22 nM. Wogonin was diluted in an MST-optimized buffer containing 1% DMSO and PBS, and Mpro was added to 10 µL of different concentrations of wogonin and thoroughly mixed. Subsequently, the mixture was filled into capillaries (NanoTemper technologies, Germany) and assayed after a 10 min equilibration at RT. The measurements were performed under auto LED power and medium MST power, and the results were plotted, with the x-axis representing the nanomolar concentrations. The dissociation constant (kd) was determined using MO Affinity analysis software v2.3 (Shen et al., 2022).

### 2.12 Surface plasmon resonance assay

Mpro was immobilized on activated COOH-sensor chips serving as the ligand on an OpenSPR system (Nicoya Lifesciences Inc., Kitchener, ON, Canada). Then, 5-40 μM wogonin was added to the sensor chip with PBS as the running buffer. The kinetic constants, including the association constant (ka), kd, and affinity (KD, KD = kd/ka), were calculated according to a 1:1 binding model.

### 2.13 Fluorescence resonance energy transfer assays for enzymatic characteristics

A fluorescent peptide with the sequence of Dabcyl-YNSTLQ↓AGLRKM-E-Edans (Ye et al., 2016) was employed as the substrate for the PEDV Mpro enzymatic and inhibition assays based on the fluorescence resonance energy transfer (FRET) effect. The 10 µM substrate peptides were incubated in 100 µL solution containing 20 mM Tris-HCl, pH 7.5, 100 mM NaCl, and10 mM CaCl_2_ at 37°C for 1 h with 0-8µg/mL purified Mpro to detect the enzymatic activity. The fluorescence emitted by the cleaved substrate was measured using the PerkinElmer VICTOR Nivo Microplate Reader (340 nm excitation, 485 nm emission). In addition, 8ug/ml Mpro was pre-incubated with 1.28nM-100µM wogonin at 37°C for 1 h, and the 10 µM substrate peptides were added to the mixture at 37°C for 1 h to test the inhibiting effect of wogonin on the PEDV Mpro. A reaction system without wogonin was used as the control. The fluorescence of the reactions was monitored according to the method mentioned above. The inhibition ratio was calculated using the following equation: percentage of inhibition (%) = 100 × [1 − fluorescence of the experimental group/the control group].

## 3 Results

### 3.1 Evaluation of wogonin cytotoxicity

**Figure 1:**
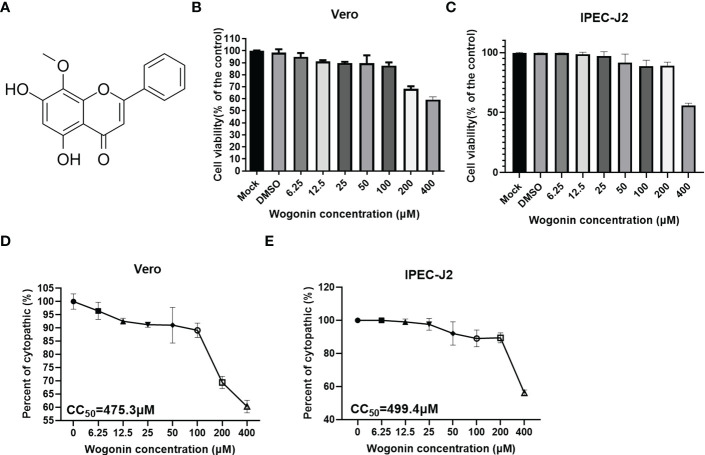
Cytotoxicity of wogonin on Vero cells. **(A)** Structure of Wogonin. **(B, C)** Evaluation of cell viability using CCK-8 assay. All the values were normalized to the control group, representing 100% cell viability. The Vero **(B)** and IPEC-J2 **(C)** cells were treated or not treated with wogonin at 48 h. Representative results of at least three independent experiments have been indicated. **(D, E)** CC50 values were calculated using GraphPad Prism software 7.0.

To determine the cytotoxicity of wogonin (**Figure 1A**) for its potential antiviral activities against PEDV, the Vero and IPEC-J2 cells were treated with gradient concentrations of wogonin in the range of 6.25-400 μM for 48 h. As shown in **Figures 1B, C**, no apparent cytotoxicity of wogonin was observed on the Vero and IPEC-J2 cells at concentrations of ≤100 μM. The CC50 of wogonin was noted to be 475.3 μM and 499.41μM (**Figures 1D, E**).

### 3.2 Antiviral activities of wogonin against PEDV

**Figure 2:**
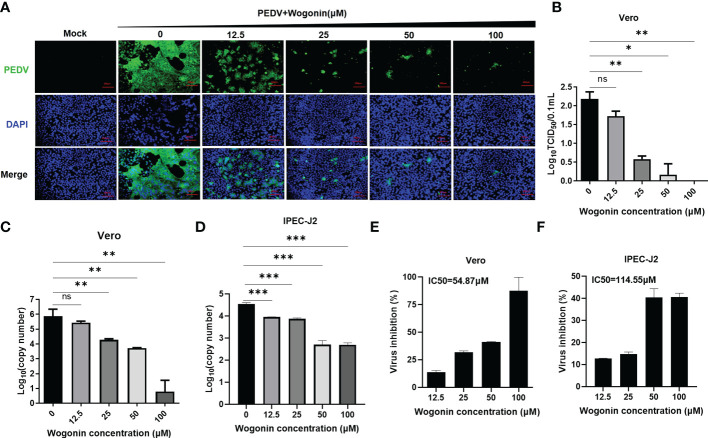
Effect of wogonin in suppressing PEDV replication in Vero cells. **(A, B)** Inhibitory effects of wogonin (12.5–100 μM) on PEDV-infected cells (MOI = 0.01) observed by IFA **(A)**. Scale bar, 200 μm. Cell lysate was harvested for TCID50 assay **(B)**. **(C, D)** Vero and IPEC-J2 cell culture supernatant were collected for RT-qPCR after incubation with serially diluted wogonin for 48 h. **(E, F)** The IC50 values were calculated using GraphPad Prism software 7.0 based on the RT-qPCR results. Bars represent the SD of triplicate trials (* denotes significant difference between test concentration and control; ns, no significant; *P < 0.05; **P < 0.01; ***P < 0.001).

To validate the antiviral activities of wogonin against PEDV, wogonin was added to PEDV-infected Vero and IPEC-J2 cells at concentrations of 12.5, 25, 50, and 100 μM, respectively, and examined by IFA. The results revealed that wogonin could inhibit PEDV proliferation until 12.5 μM in Vero cells (**Figure 2A**). Furthermore, the TCID50 assay was performed, and the dose-dependent inhibition of PEDV in Vero cells was determined (**Figure 2B**). Notably, the IC50 value of wogonin (58.87 μM and 56.56 μM) was far lower than its CC50 value (475.3 μM and 499.4 μM) obtained in the Vero (**Figures 2C, E**) and IPEC-J2 cells (**Figures 2D, F**), which indicated the ability of wogonin to suppress PEDV replication with only slight cytotoxicity. Moreover, the therapeutic index of wogonin was 8.0 and 8.8μM in IPEC-J2 cells. These findings revealed that wogonin could inhibit PEDV propagation in a dose-dependent manner, exhibiting enhanced antiviral activity with increasing wogonin concentration.

### 3.3 Effect of wogonin on different stages of PEDV life cycle

**Figure 3:**
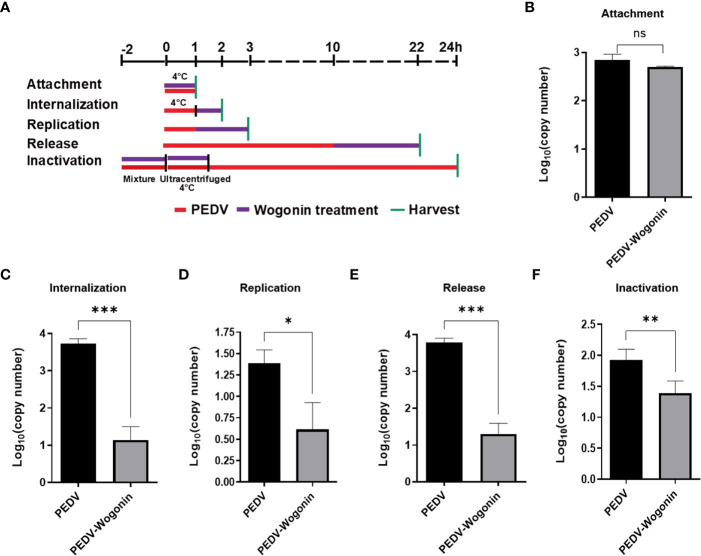
Effect of wogonin on different stages of the PEDV life cycle in Vero cells. **(A)** Simplified schematic representation of PEDV life cycle. **(B-F)** PEDV-infected cells were treated with wogonin during **(B)** viral attachment, **(C)** internalization, **(D)** replication, **(E)** release, and **(F)** inactivation stage. PEDV replication was determined by RT-qPCR. Values represent mean ± SD for three independent experiments. Ns indicates no significance, *P < 0.05; ** *P* < 0.01; ***P < 0.001.

To understand the precise mechanism of the inhibitory effect of wogonin on PEDV propagation, the influence of wogonin on the PEDV at different stages of infection was assessed. The Vero cells were treated with 50 μM of wogonin during different stages of the PEDV life cycle (0.05 MOI) and then harvested to assay the viral RNA (**Figure 3A**). In the viral attachment assay, no significant difference in the level of PEDV RNA was observed between the wogonin-treated infected cells and the untreated infected cells (**Figure 3B**). However, wogonin significantly inhibited PEDV internalization, replication, and release (**Figures 3C-E**). In addition, wogonin interacted with PEDV particles, as the pre-exposure of the virus to wogonin altered the infectivity of PEDV particles (**Figure 3F**).

### 3.4 Mechanism of the inhibitory effect of wogonin on PEDV replication

**Figure 4:**
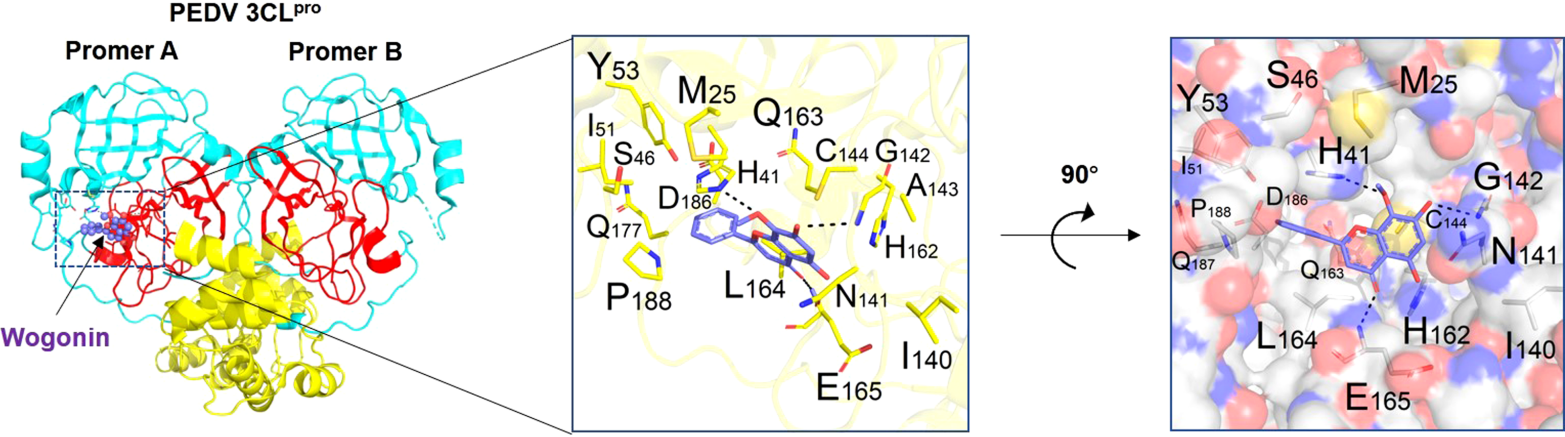
Binding mode analysis of wogonin and Mpro. Two perpendicular views are shown. Mpro is indicated as ribbon (yellow) and cylinder (denotes residues, green). Wogonin is shown in ball-and-stick models (purple). Protomer A (catalytically competent enzyme) is presented on the left and protomer B (inactive enzyme) is shown on the right. Domain I (residues 8−101), domain II (residues 102−184), and domain III (residues 201−306) can be seen.

Binding affinity analysis (−10.0 kcal/mol) and binding mode examination suggested that Mpro can favorably combine with wogonin. This binding between Mpro (protein) and wogonin (ligand) was stable because the ligand was embedded in the groove of the active pocket owing to its small size. **Figure 4** shows the optimal docked model of Mpro with wogonin. The ligand formed hydrogen bonds with E165, H41, and G142 residues, and the hydrophobic interactions of M25, Y53, I51, I140, C144, Q163, L164, and P188 also had a non-negligible role in the stabilization of wogonin. Thus, wogonin inhibited PEDV replication by blocking the binding pocket of Mpro.

### 3.5 Binding affinity of wogonin to Mpro

**Figure 5:**
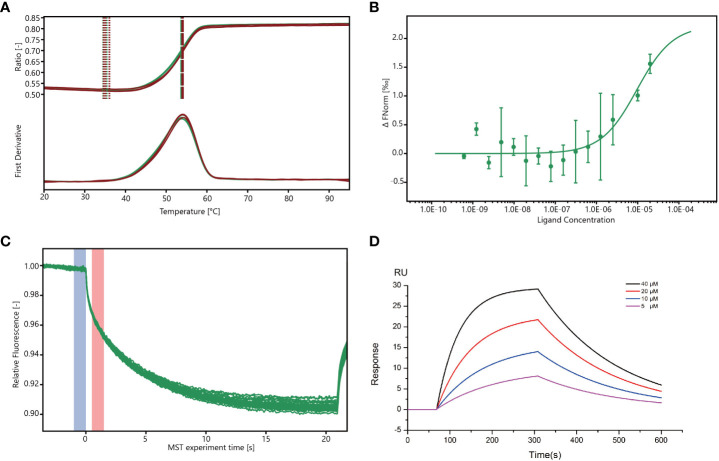
Wogonin blocks Mpro to disrupt PEDV infection. **(A)** Stability of Mpro determined by DSF assay. For the DSF assay, 1 mg/mL Mpro and 40 mM wogonin were employed. **(B, C)** Binding of Mpro to wogonin was investigated by MST assay. For MST assay, 40µM–1.22 nM wogonin and 200 nM Mpro were employed. **(D)** Binding curve of wogonin with Mpro in surface plasmon resonance (SPR) assay.

The stability of Mpro was analyzed using a differential scanning fluorimetry (DSF) assay (Wright et al., 2017), and the results showed that the melting temperature (*T*m) of Mpro (53.8°C) was similar to that of Mpro co-cultured with wogonin (54°C), thus suggesting that wogonin did not affect Mpro stability (**Figure 5A**). In addition, the microscale thermophoresis (MST) assay used to validate the interaction between wogonin and Mpro and quantify the binding affinity revealed that wogonin showed positive Mpro binding with a dissociation constant (kd) value of 9.589E-06 (± 6.917E-06) (**Figures 5B, C**). Furthermore, the investigation of the binding affinity between Mpro and wogonin through the use of a surface plasmon resonance (SPR) assay (**Figure 5D**) indicated a significant and dose-dependent increase in the SPR signal due to wogonin, and the affinity constant for the interaction between wogonin and Mpro was 16 μM. Overall, these results confirmed that wogonin could interact with Mpro to disrupt PEDV infection.

### 3.6 Wogonin inhibits PEDV Mpro enzymatic activity

**Figure 6:**
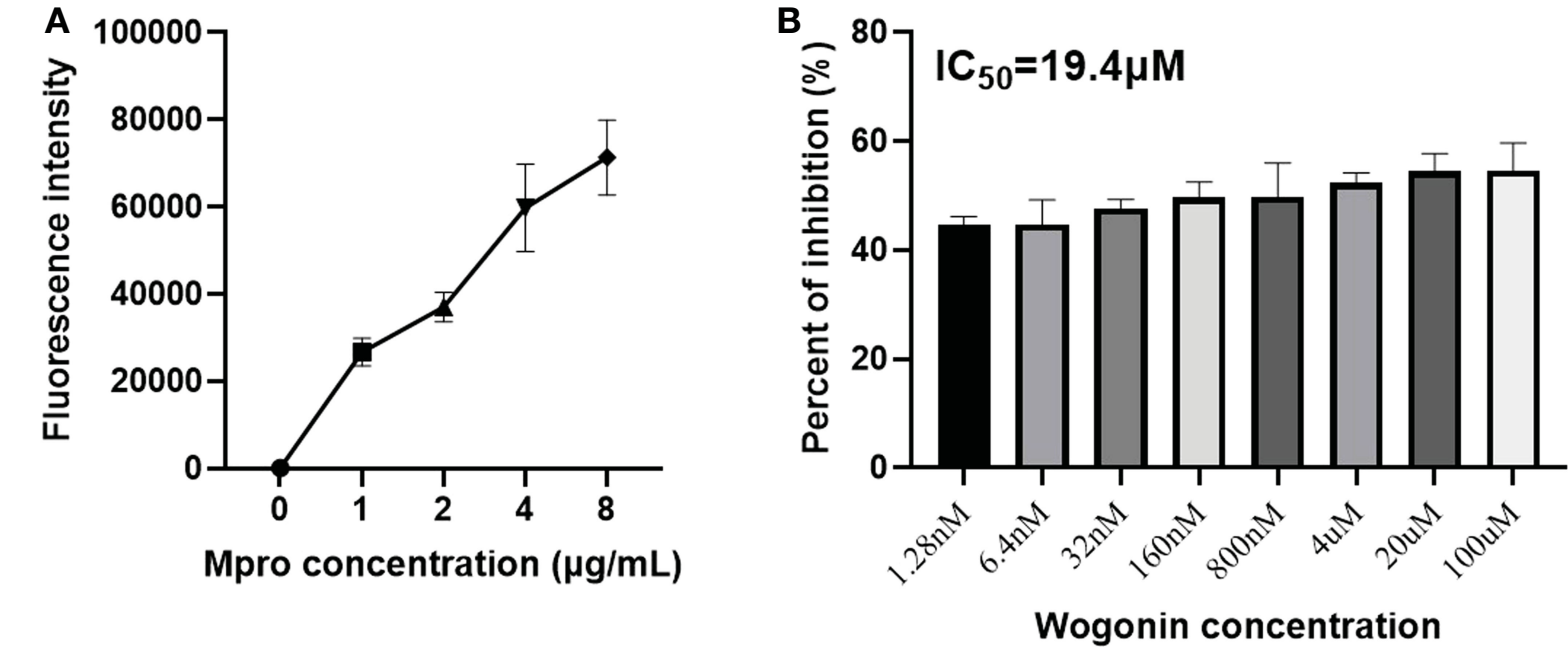
Inhibition of the PEDV Mpro enzymatic activity by wogonin. **(A)** The fluorescence resonance energy transfer (FRET) assay was performed to analyze the enzymatic activity of the purified PEDV Mpro. **(B)** The effect of wogonin on the activity of Mpro by FRET assay. IC50 was determined using GraphPad Prism 6 software. Data are shown as the means ± SD of three independent experiments.

To confirm the inhibition of the PEDV Mpro *in vitro*, we performed an enzymatic inhibition assay, which specifically measures protease activity. Fluorescence increased with increasing Mpro into the substrate (**Figure 6A**), suggesting that our purified Mpro protein could effectively cleave the fluorogenic peptide substrate. In addition, the inhibition activity assay showed that wogonin results in a concentration-dependent inhibition of PEDV Mpro activity, with an approximately 54% inhibition of protease activity at 100 μM, and the IC50 was 19.4 μM (**Figure 6B**).

## 4 Discussion

Currently, no effective commercial treatment measures against PEDV infection exist, and there is an urgent need to develop prophylactic and therapeutic technologies. Traditional Chinese medicine, which presented remarkable efficacy in treating COVID-19 infection, has attracted much attention as an effective antiviral therapy (Ren et al., 2021), and the selection of herbal medicines and the screening of effective antiviral active substances have become a major challenge. Here, wogonin exhibited excellent antiviral activity against a PEDV variant isolate, interacting with the PEDV particles and inhibiting the internalization, replication, and release of PEDV. The protective effects of bioactive compounds in wogonin against infection of multiple viruses have been evidenced in certain cases, such as respiratory syncytial virus (RSV), vesicular stomatitis virus (VSV) and varicella-zoster virus (VZV)(Ma et al., 2002; Blach-Olszewska et al., 2008; Gould, 2014). The results of the present study provide evidence that wogonin inhibits the cleavage activity of Mpro, which might explain its effect on viral RNA synthesis. Additionally, wogonin may directly inactivate PEDV by disrupting the integrity of virions. It was reported that a boiled extract of wogonin enables the interception of viral activity (Li-Weber, 2009). Wogonin might interpose the virus–cell membrane fusion of PEDV through spike protein (S), inhibiting viral internalization (Liu et al., 2022). Additionally, the effect on PEDV release may also be mediated by the modulation of innate immune responses (Ma et al., 2002). Wogonin triggers IFN-induced antiviral signaling (STAT1/IRF3 pathway) and activates anti-inflammatory responses *via the* modulation of NF-κB/AP1/MAPK signaling pathways (Blach-Olszewska et al., 2008; Moreira et al., 2016; Chu et al., 2020). Wogonin shares structural similarities with kaempferol, quercetagetin, and chrysin, which are flavonoids with strong inhibitory activities against CoV (Dai et al., 2020). Investigating the inhibitory effects of current antiviral drugs against the replication and cytotoxicity of PEDV in Vero cells revealed that the IC50 of 3-(aminocarbonyl)-1phenylpyridinium and 2,3-dichloronaphthoquinone were 28.63 and 38.45 μM, respectively, and their corresponding CC50 were 73.8 and 21.79 μM (Zhou et al., 2021). In contrast, wogonin showed the highest therapeutic index (8.07) for PEDV, with an IC50 of 58.87 μM and a CC50 of 475.3 μM. These findings suggest that wogonin possesses stronger inhibitory effects against PEDV, providing useful insights for the design and optimization of small molecule inhibitors targeting PEDV.

Small molecule inhibitors must form efficient and stable complexes to achieve the maximum inhibition of viral replication (Yin et al., 2007). The viral proteases of CoVs play essential roles in CoV replication and are commonly recognized as ideal active sites in antiviral drug research. Wogonin has been reported to have a good affinity with SARS-CoV-2 Mpro and mediates inflammation and immunoregulation through the MAPK signal pathway (Sheng et al., 2022). Targeting the conserved Mpro active site *via* potent inhibitor molecules can not only hinder the involvement of the protein in viral replication but can also prohibit the protein from interfering with the host’s innate immune response against viral invasion (Yang et al., 2006). The results of the present study provide evidence of a strong interaction between wogonin and Mpro; however, further animal experiments are needed to verify its therapeutic effects on PEDV-infected piglets. The Mpro forms a dimer with two promoters (denoted as “A” and “B”) oriented almost at right angles to each other. A previous study demonstrated that the substrate-binding site of Mpro is located in a cleft between domain I (residues 8−101) and II (residues 102−184) (Hsu et al., 2005; Ye et al., 2016). Interestingly, the active site of SARS-CoV Mpro contains Cys145 and His41, while that of Mpro contains Cys144 and His41 residues, creating a similar catalytic dyad in which the cysteine functions as a common nucleophile in the proteolytic process (Grum-Tokars et al., 2008; Dai et al., 2020; Ye et al., 2020; Zhou et al., 2021). The docking analysis results obtained in the present study demonstrated that wogonin formed hydrogen bonds with residues E165, H41, and G142 of Mpro and hydrophobic interactions with Q163 and L164. It has been reported that Glu165 consists of a specificity binding pocket (Anand et al., 2003) and that Cys144, Glu165, and Gln191 in Mpro are conserved (Mukherjee et al., 2008; Li et al., 2020). Thus, theoretically, wogonin can block the substrate combination and catalysis process, thereby inhibiting the enzyme’s activity, which is consistent with the binding sites of previously reported compounds (GC376, 2,3-dichloronaphthoquinone, and quercetin). Furthermore, the FRET assay was performed to verify the inhibiting effect of wogonin on PEDV Mpro, and it was found that wogonin could effectively inhibit PEDV Mpro activity.

Although most antiviral drugs can directly inhibit viral replication by targeting specific viral proteases, they gradually lose their efficacy owing to rapid viral mutation. Iketani et al. used serial passages to analyze the drug resistance characteristics of SARS-CoV *in vitro* and found that the Mpro mutation of SARS-CoV led to few drug options, with the drugs developed against the virus encountering a high drug resistance barrier (Iketani et al., 2022). Hence, compounds targeting Mpro mutations provide important references for the development of medicines, specifically for CoV infection. Wogonin reverses drug resistance in cancer cells by overexpressing inflammation-associated dihydrodiol dehydrogenases or targeting oncogenic transcription factors (Xu et al., 2014; Rajagopal et al., 2018). However, the mechanism of antiviral resistance in terms of wogonin is rarely reported. Traditional Chinese medicine can reduce the adverse reactions of antiviral drugs, improve medication compliance and antiviral effects, and reduce the influencing factors related to the occurrence of drug resistance. Recently, the goal of reducing drug resistance can be achieved by reducing the factors related to drug resistance in patients undergoing antiviral therapy (Han et al., 2016). Wogonin, as the therapeutic adjunct of an antiviral, should also be achieved in drug-resistant antiviral therapy by reducing resistance-related factors.

Root extracts of wogonin, one of the most popular traditional Chinese medicines, have been widely used for thousands of years. Extracts in the form of tablets or capsules/drops are commercially available (Huynh et al., 2017). Experimental and clinical evidence has demonstrated that wogonin exhibits antioxidant, anti-inflammatory, and neuroprotective activities and can be used in the treatment of bacterial and viral infections (Huynh et al., 2020). Zhao et al. examined the *in vivo* impact of wogonin on fetal development in pregnant mice and found that an intravenous dose of 40 mg·kg^-1^ leads to maternal weight gain and affects fetus development significantly, including body weight, resorption, the live birth index, fetal skeletal alterations, and fetal genotoxicity (Zhao et al., 2011). However, the newly synthesized flavonoids derived from wogonin have shown their safety and application potential for use as anticancer drugs *in vitro* and *in vivo* (Chen et al., 2010). Thus, upon the confirmation of anti-PEDV in newborn piglets, the wogonin concentration should be fine-tuned to obtain the desired efficiency in pregnant sows and newborn piglets. Although, many researchers have reported that there has been an enhanced chemo-sensitization effect on drug-resistant cancer cells following the treatment with wogonin as an adjuvant at appropriate doses (Xu et al., 2014). However, whether wogonin could improve current vaccination strategies needs to be further explored.

In summary, wogonin exerted excellent inhibitory effects against PEDV variant strains by targeting the viral Mpro and has significant potential to produce strong therapeutic effects on animals. Exploiting the backbone structures of these molecules could further help in the development of drugs with higher affinity and specificity for Mpro. In addition, the active site of Mpro must also be further explored for the development of novel drugs.

## Data availability statement

The original contributions presented in the study are included in the article/supplementary material. Further inquiries can be directed to the corresponding authors.

## Author contributions

JW, DY and XP conceived and designed the experiments. JW, DY, LY, XS and XZ were responsible for sampling and sample testing. XZ, FX and XP analyzed the data. JW, DY, LY, XS, FX and XZ wrote the paper. XP, and YD edited the paper. All authors contributed to the article and approved the submitted version.
